# Serums and Vaccines1Read before the Elmira Academy of Medicine, December, 1910

**Published:** 1911-03

**Authors:** Charles Haase

**Affiliations:** Elmira N. Y. 110 West Church Street


### Serums and Vaccines[1]

By CHARLES HAASE, M.D.,
Elmira N. Y.

A VAST amount of work has been done recently in developing serum and especially vaccine therapy, and the progress made shows that we have valuable aids with which to fight diseases due to bacteria. We are getting a little nearer, and some

1. Read before the Elmira Academy of Medicine, December, 1910

day may realize the dream of the immortal Pasteur when he said, "It is possible for man to eradicate every contagious disease from the face of the earth."

Jenner with his inocculation for the prevention of smallpox, Pasteur with his virus for the prevention of hydrophobia, Koch with his tuberculin, and Behring with his diphtheria antitoxin were pioneers in this field, but it was Sir A. E. Wright introducing his opsonic theory and his small infrequent injections of sterile bacterial vaccines that gave the great impetus to this form of therapy.

### SERUMS.

The antiserums or antitoxins produce a passive immunity, that is, they neutralise the toxins formed by the bacteria, and the immunising forces of the body destroy the pathogenic germs.

The first practical step in serum therapy was made when it was discovered that the bouillon in which diphtheria bacilli grew contained a soluble toxin. It was discovered that a measured quantity of this filtered bouillon would kill a guineapig of a certain weight. Next it was discovered that when less than a fatal dose was injected, after a time the animal developed a resistance to a dose that would kill an untreated animal. The injection of the non-fatal dose of toxin had caused the production of an antitoxin.

Diphtheria antitoxin has greatly reduced the mortality rate from diphtheria. It is not only curative, but it is a valuable preventative. It has no direct action on the diphtheria bacillus, in fact, this serum is said to be a good culture medium for growing the Klebs-Löffler bacillus. The discovery of diphtheria antitoxin led to the hope that similar sera could be produced for other infectious diseases, but it was discovered that the other bacteria did not produce soluble toxins in quantity. The only other important exception was the tetanus bacillus. Tetanus antitoxin is very valuable, especially as a preventative.

Flexner's antimeningococcic serum belongs in a class by itself. It is claimed to be antitoxic, antibacterial, and opsonofying. It is made by injecting into the horse: first increasing doses of killed meningococci, then increasing doses of living meningococci, and finally increasing doses of extract of meningococci.

Some have claimed good results from Marmorek antistreptococcic serum in scarlatina, erysipelas, abscesses, puerperal fever, pyemia, and tuberculosis with mixed infection. Antistreptococcic and antistaphylococcic serums are of little value because the streptococcus and staphylococcus depend for their activity, in great part, not upon soluble toxins, but upon endotoxins which are in the bodies of the bacteria. In these cases there is the additional work of destroying the bacteria and serums take no part in this work.

Yersin's bubonic plague serum is said to be of great value.

Frazer has produced an antivenomous serum which gives satisfactory results if used immediately after a snake bite.

Lustigo's antirabic serum seems to give better results than Pasteur's vaccine in the treatment of hydrophobia.

Serums are sometimes used in making diagnosis: as, the serum from a typhoid patient in making a Widal test or the serum from a syphilitic in making Wasserman's reaction.

One attack of certain diseases will stimulate the specific immunising forces, so that the body is protected from a second attack of the specific disease. This is due to the presence in the blood and tissue juices of opsonins, agglutinins, bacteriolysins, and other antibodies.

Every individual has a relative quantity of immunity. It may be high or low. When a body is in a state of low immunity or low resistance there is a deficiency in opsonins. Opsonins (opsono—I prepare for food) are substances that sensatise the bacteria so that they can be engulfed and destroyed by the phagocytes.

In many cases the natural immunising process is too sluggish to prevent an infection spreading, and must be aroused by artificial means. Wright demonstrated that if a proper number of sterile pathogenic germs be introduced under the skin, the immunising mechanism is aroused so that the pathogenic bacteria and its products are destroyed. This increased germ destroying power continued from five to ten days or more after the inoculation, and that if the inoculations were repeated at proper intervals a permanent immunity would be established. He also measured quantitatively the opsonins, and was able to raise the opsonic index by injecting increasing doses of bacterial vaccines. Fortunately this elaborate technic of estimating the opsonic index is essential only in exceptional cases.

A bacterial vaccine consists of the specific organism isolated in pure culture, and after sterilisation by heat, suspended in normal salt solution. There are two varieties of vaccines. The antogenous which is grown from bacteria obtained from the patient, and it has the advantage that it is made up of the same strain of bacteria that is producing the disease. Stock vaccines are made from bacteria obtained from other sources than the patient. They have the advantage that they can be used at once, as it is not necessary to wait for the preparation of a stock vac cine. They can be kept on hand.

To prepare a vaccine, germs are grown upon nutrient agar or a blood serum medium in an incubator at 37 degrees C., and should be used when twelve to twenty-four hours old. They are washed off the culture medium with 0.87 per cent. sodium chloride solution and thoroughly shaken to break up the colonies. A small portion of this emulsion is mixed with an equal portion of normal blood, spread upon a slide, fixed, stained and the number of red cells and bacteria, in a number of fields, counted. From these figures the number of bacteria per cubic millimeter can easily be computed as we know we have 5,000,000 red corpuscles in that quantity. The emulsion is kept at 60 degrees C. in a sealed glass tube for one hour to kill the bacteria. A small quantity is then incubated at 37 degrees C. for twenty-four hours to determine the absence of living organisms. The remainder is diluted to the desired strength with normal salt solution, to which 0.25 per cent. of carbolic acid has been added, and kept in rubber capped bottles.

Wright believes that a proper dose of vaccine should cause a period of intoxication (negative phase) followed by a stimulation of the immunising forces (positive phase). By gradually increasing the dose the immunising forces are progressively increased so that they overpower the infection. The ideal immunising response can be produced by slurring over the negative phase or by making it so mild that it can not be observed clinically.

It is thought that in local infections the bacteria and toxins that stimulate the immunising forces are not absorbed, owing to an obstruction of the lymphatics and capillaries. Drainage, hyperemia, massage and passive movements aid in bringing the toxins and bacteria in contact with the immunising forces. Vaccine therapy does not do away with the necessity of intelligent surgery.

The limitations of vaccine therapy, as Wright sees them, are: (1) vaccine therapy can be applied only where an exact and complete bacteriological diagnosis can be made, and where the diagnosis is kept up to date; (2) it can be applied only by those who have some acquaintance with bacteriology, some understanding of the rationale of vaccine therapy, and a knowledge of the proper dose of the particular vaccine which it is proposed to employ; (3) a limit is placed on the efficacy of inoculations by the fact that there are definite limits to the responsive power of the patient; (4) successful results can be obtained only where an efficient lymph stream can be conducted through the foci of infection; (5) in long-standing infections vaccine therapy can give definite results only after a long succession of inoculations; (6) in some cases it is essential to success that the dose of the vaccine be controlled by the opsonic index.

USE OF VACCINES.

The staphylococcic vaccines have given the best results. Furuncles, carbuncles, acne, felons, sties, ostitis, periostitis, osteomyelitis, chronic sinuses, empyemia, secondary infection of lungs, sycosis, impetigo, and septic wounds have all been successfully treated with staphylococcic vaccines. There are three varieties, staphylococcus aureus, albus, and citreus. The dose ranges from 2 to 500 million.

Streptococcic vaccines have proven of value in scarlatina, erysipelas, septic endocarditis, and in some cases of pyemia and puerperal sepsis. Some clinicians have obtained good results in amygdalitis, acute articular rheumatism following infection of tonsils, middle ear, and mastoid infections. On account of the numerous strains of streptococci an antogenous vaccine should be prepared. Dose 10 to 50 million.

Bacillus coli vaccines have given good results in certain cases of cystitis, cholecystitis, appendicitis, and in abscesses following operations involving the intestines. Dose 5 to 50 million.

Gonococcic vaccines do good in some cases of chronic gonorrhea and gonorrheal rheumatism. In acute cases the results have been disappointing.

Typhoid vaccines are very valuable for the prevention of typhoid fever. In our army among 10,000 inoculated men there developed only 45 cases of typhoid fever with 4 deaths; while 272 cases arose with 46 deaths among 8,000 not inoculated. Nearly all cases in the inoculated men occurred in those who had but one injection, and all 4 deaths were among them. Three inoculations, at ten days' interval, are given. The first dose is 500 million typhoid bacilli and at the second and third inoculations twice this number.

Pneumococcic vaccines have proven of value in the hands of a few clinicians. Dose 5 to 50,000 million. Tuberculin or tubercle bacilli vaccine has given good results in some cases of tubercular infections of the glands, bones, joints, and in early or subacute cases of pulmonary involvement. It is a valuable aid in diagnosis. Dose of tuberculin, 1-2000 of a milligram cautiously increased.

Vaccines from other bacteria are being tried, but no definite conclusions as to their value have been obtained.

110 West Church Street.

---

E. M. Foote of New York, says that in an emergency an exploring needle may be used for tapping the pleural cavity, the patient so placed that the serum will all drip away without using an aspirator.—*Medical Record,* February 5, 1910.

